# Protective role of microRNA-29a in denatured dermis and skin fibroblast cells after thermal injury

**DOI:** 10.1242/bio.014910

**Published:** 2016-01-21

**Authors:** Jie Zhou, Xipeng Zhang, Pengfei Liang, Licheng Ren, Jizhang Zeng, Minghua Zhang, Pihong Zhang, Xiaoyuan Huang

**Affiliations:** Department of Burns and Plastic Surgery, Xiangya Hospital, Central South University, Changsha, Hunan 410008, People's Republic of China

**Keywords:** Denatured dermis, Fibroblast cells, MicroRNA-29a, Thermal injury, Wound healing

## Abstract

Our previous study has suggested that downregulated microRNA (miR)-29a in denatured dermis might be involved in burn wound healing. However, the exact role of miR-29a in healing of burn injury still remains unclear. Here, we found that expression of miR-29a was notably upregulated in denatured dermis tissues and skin fibroblast cells after thermal injury, and thereafter gradually downregulated compared with control group. By contrast, the expression of collagen, type I, alpha 2 (COL1A2) and vascular endothelial growth factor (VEGF-A) were first reduced and subsequently upregulated in denatured dermis tissues and skin fibroblast cells after thermal injury. We further identified COL1A2 as a novel target of miR-29a, which is involved in type I collagen synthesis, and showed that miR-29a negatively regulated the expression level of COL1A2 in skin fibroblast cells. In addition, VEGF-A, another target gene of miR-29a, was also negatively mediated by miR-29a in skin fibroblast cells. Inhibition of miR-29a expression significantly promoted the proliferation and migration of skin fibroblast cells after thermal injury, and knockdown of COL1A2 and VEGF-A reversed the effects of miR-29a on the proliferation and migration of skin fibroblast cells. Furthermore, we found that Notch2/Jagged2 signaling was involved in miR-29a response to burn wound healing. Our findings suggest that downregulated miR-29a in denatured dermis may help burn wound healing in the later phase, probably via upregulation of COL1A2 and VEGF-A expression, which can further enhance type I collagen synthesis and angiogenesis.

## Abstract (precis)

**Summary:** Inhibition of miR-29a can promote the proliferation and migration of skin fibroblast cells after thermal injury, and upregulate the production of COL1A2 and VEGF-A to further enhance the collagen synthesis and angiogenesis in skin and help burn wound healing in the later phase.

## INTRODUCTION

Our previous studies have reported that preservation of the denatured dermis when performing large sheets of split thickness skin grafting shows satisfactory clinical effects for the treatment of the deep burn wound, as denatured dermis can help lessen scar contracture, as well as improve appearance and function (Huang, 2009; Liu et al., 2005; Yang et al., 2005). However, the molecular mechanism by which the denatured dermis plays a role in structural remodeling during wound healing has never been reported.

MicroRNAs (miRs) are a class of 18-25 nucleotide non-coding RNAs. It has been well established that miRs can directly bind to the 3′-untransled region (UTR) of their target mRNAs, leading to mRNA degradation or inhibition of protein translation (John et al., 2004). Growing evidence indicates that miRs are involved in the regulation of cell survival, proliferation, differentiation, and migration, through mediating the expression of their target genes (Ambros, 2004). Our previous study has compared profiled miR expression between the denatured dermis after burn injury and the paired normal skin, and showed that 66 miRs were differentially expressed in denatured dermis compared to paired normal skin, among which 32 were upregulated and 34 were downregulated (Liang et al., 2012). We have found that downregulation of miR-23b dramatically promoted the proliferation and migration of heat-denatured fibroblasts by activating the Notch1 and TGF-β signaling pathways (Zhang et al., 2015). Among these differentially expressed miRs, the level of miR-29a was significantly decreased in denatured dermis at day 4 after burn, suggesting that miR-29a may be associated with the protective role of denatured dermis in the healing of burn injury (Liang et al., 2012).

In fact, several predicted targets of miR-29a have been found to participate in tissue remodeling and wound healing. For instance, vascular endothelial growth factor (VEGF)-A has various effects, including mediating increased vascular permeability, inducing angiogenesis, vasculogenesis and endothelial cell growth, promoting cell migration, and inhibiting apoptosis, and thus plays a crucial role in tissue repair and wound healing (Eming and Krieg, 2006; Ferrara, 2009). Recently, VEGF-A has been identified as a direct target of miR-29a (Chen et al., 2014; Yang et al., 2013). Moreover, Yang et al. (2013) showed that social isolation delayed oral mucosal healing, and that isolated rats persistently exhibited lower VEGF-A levels, partially at least due to a higher level of miR-29a However, the exact role of miR-29a in healing of burn injury as well as the underlying mechanisms remains largely unclear.

In the present study, we examined the expression of miR-29a in denatured dermis tissues and skin fibroblast cells after thermal injury. Moreover, we investigated the role of miR-29a on the proliferation and migration of skin fibroblast cells after thermal injury. In addition, we also identified novel target genes of miR-29a and explored the related signaling pathway associated with tissue remodeling in skin after thermal injury.

## RESULTS

### Expression of miR-29a in denatured dermis tissues after thermal injury

**Fig. 1.**
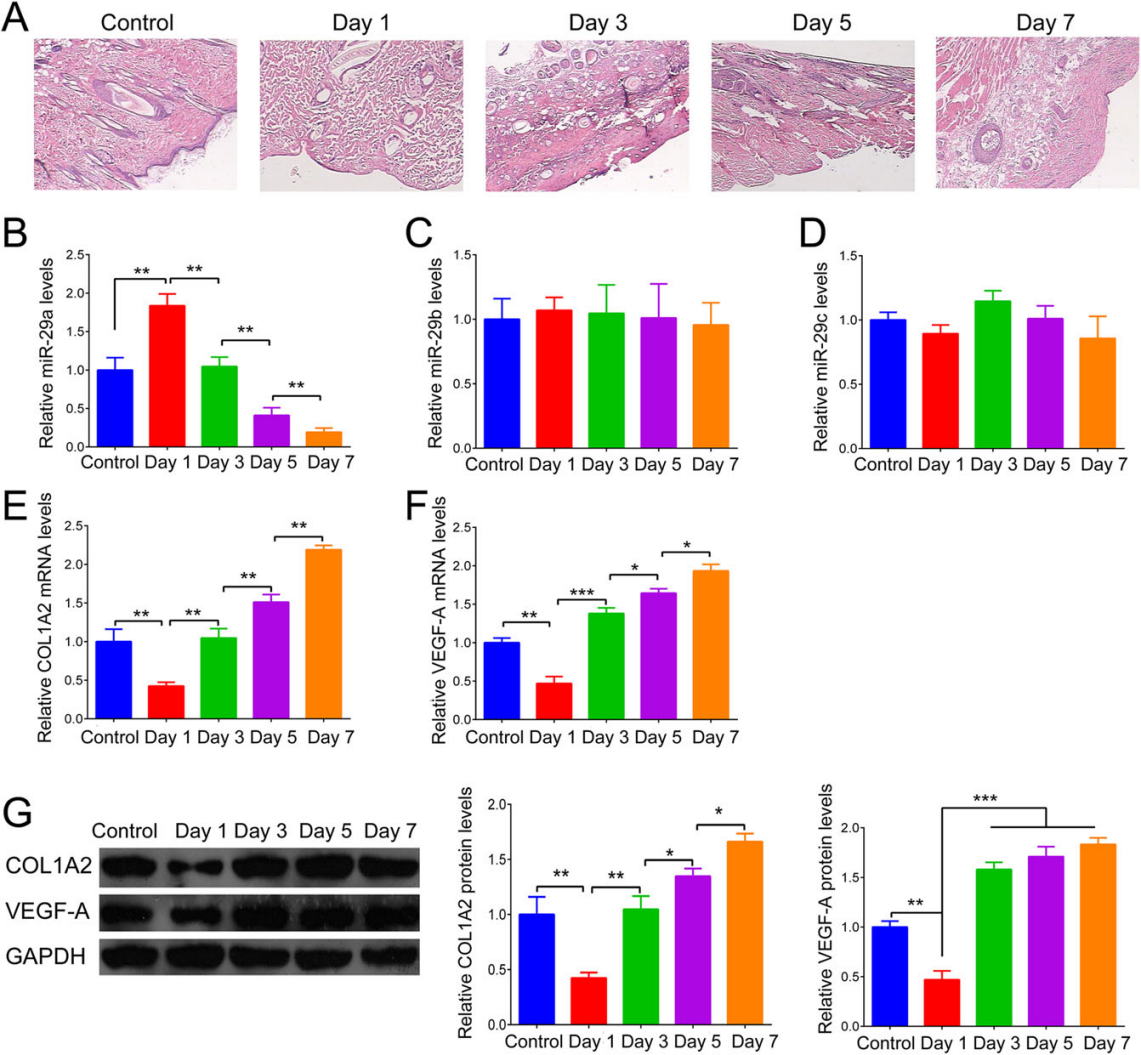
**The expression of miR-29a, COL1A2 and VEGF-A in denatured dermis tissues after thermal injury.** (A) Representative images of HE staining for the denatured dermis of rats at different time points after thermal injury. Control: the dermis tissues of rats without thermal injury. (B-D) After thermal injury, real-time RT-PCR was performed to examine the relative expression of miR-29a (B), miR-29b (C), and miR-29c (D) in denatured dermis tissues of rats at different time points. (E-F) The relative mRNA levels of *COL1A2* (E) and *VEGF-A* (F) were also detected. Control: rats received sham injury. (G) Western blot was performed to quantify the relative protein levels of COL1A2 and VEGF-A in denatured dermis tissues of rats at different time points after thermal injury. *P<0.05, **P<0.01 vs Control. The experiments were independently repeated for three times. Data are presented as mean±s.d.

**Fig. 2.**
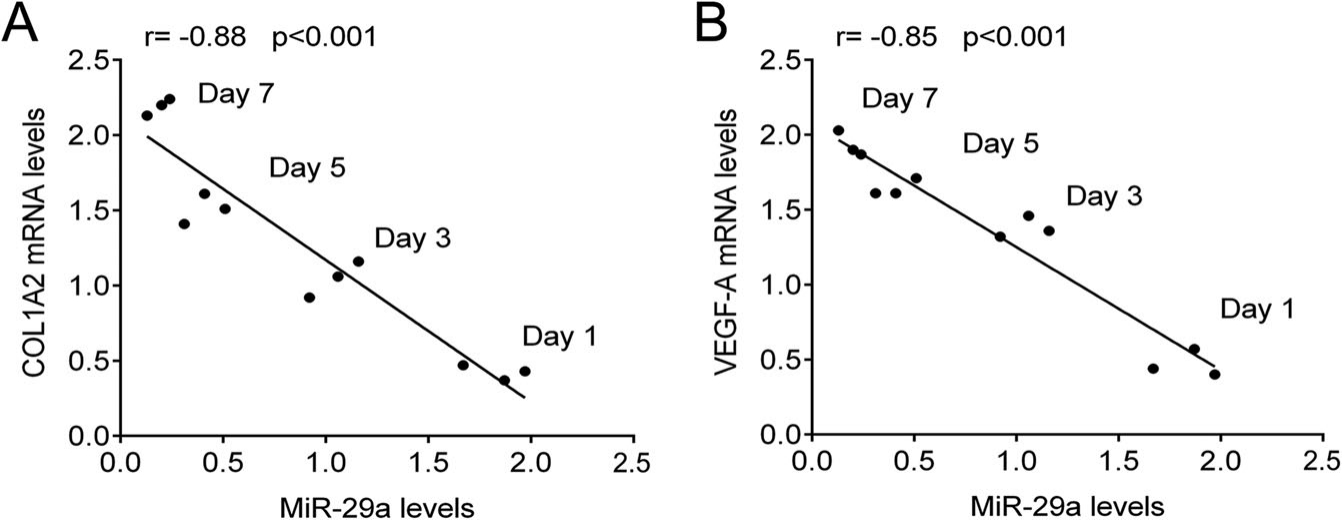
**miR-29a was negatively correlated with COL1A2 and VEGF-A.** Pearson correlation analysis was used to analyze the relationship between miR-29a and COL1A2 (A), and between miR-29a and VEGF-A (B).

We first constructed a rat model of thermal injury. Denatured dermis tissues were isolated at days 1, 3, 5 and 7 after thermal injury and then used to perform HE staining (Fig. 1A). To preliminarily reveal the role of miR-29 in denatured dermis, we examined expression levels of the miR-29 family including miR-29a, miR-29b and miR-29c in denatured dermis of rats at different time points after thermal injury. As shown in Fig. 1B, miR-29a was significantly upregulated at day 1 after thermal injury, but gradually downregulated. At day 7 after thermal injury, the expression of miR-29a was only 20% that of rats in the control group. However, the expression levels of miR-29b and miR-29c were comparable at different time points (Fig. 1C,D). We further determined the mRNA and protein levels of COL1A2 and VEGF-A in denatured dermis of rats at different time points after thermal injury. As shown in Fig. 1E-G, the mRNA and protein levels of COL1A2 and VEGF-A were significantly downregulated at day 1 after thermal injury, but gradually upregulated. Importantly, we analyzed the correlation between miR-29a and COL1A2, and miR-29a and VEGF-A. As shown in Fig. 2A,B, miR-29a was negatively correlated with COL1A2 and VEGF-A at mRNA levels.

### Expression of miR-29a, COL1A2 and VEGF-A in BJ cells after thermal injury

**Fig. 3.**
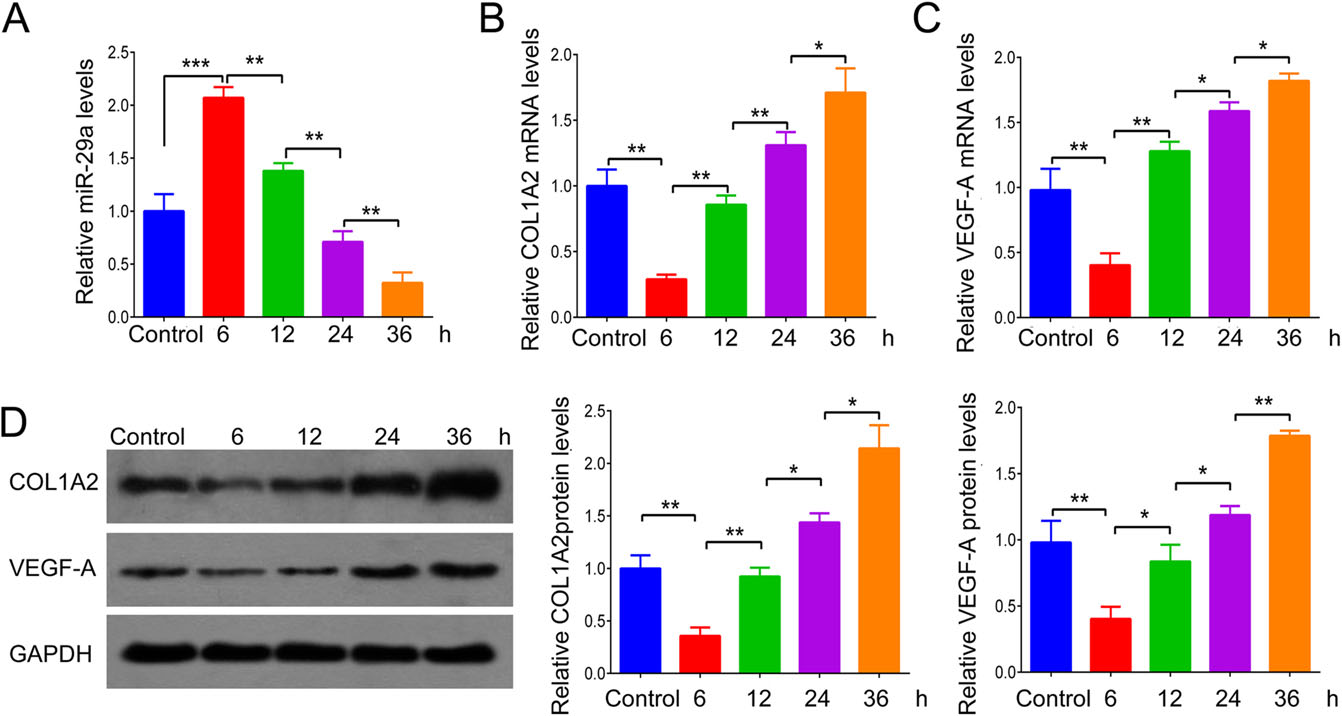
**The expression of miR-29a, COL1A2 and VEGF-A in BJ cells after thermal injury.** (A) Real-time RT-PCR was performed to examine the relative miR-29a expression in human skin fibroblast BJ cells at different time points after thermal injury. (B,C) Real-time RT-PCR was performed to examine the relative mRNA levels of *COL1A2* (B) and *VEGF-A* (C) in human skin fibroblast BJ cells at different time points after thermal injury. (D) Western blot was performed to quantify the relative protein levels of COL1A2 and VEGF-A in human skin fibroblast BJ cells at different time points after thermal injury. Control: BJ cells received sham injury. *P<0.05, **P<0.01, ***P<0.001 vs Control. The experiments were independently repeated for three times. Data are presented as mean±s.d.

We further determined the expression of miR-29a, COL1A2 and VEGF-A in BJ cells after thermal injury. Fig. 3A shows the expression of miR-29a in skin fibroblast BJ cells at 6, 12, 24 and 48 h after thermal injury. Similar findings were also observed in that the expression level of miR-29a was increased shortly after thermal injury but gradually reduced 24 h after thermal injury, compared to the control group. In addition, it has been demonstrated that VEGF-A is a target gene of miR-29a (Chen et al., 2014), and it plays an important role in angiogenesis and tissue remodeling (Yang et al., 2013). As fibroblasts are the main source of VEGF-A, we further determined the expression of VEGF-A in BJ cells after thermal injury. The mRNA and protein levels of COL1A2 and VEGF-A were notably downregulated shortly after thermal injury but gradually upregulated 24 h after thermal injury, when compared to the control group (Fig. 3B-D).

### COL1A2 and VEGF were negatively mediated by miR-29a in BJ cells

**Fig. 4.**
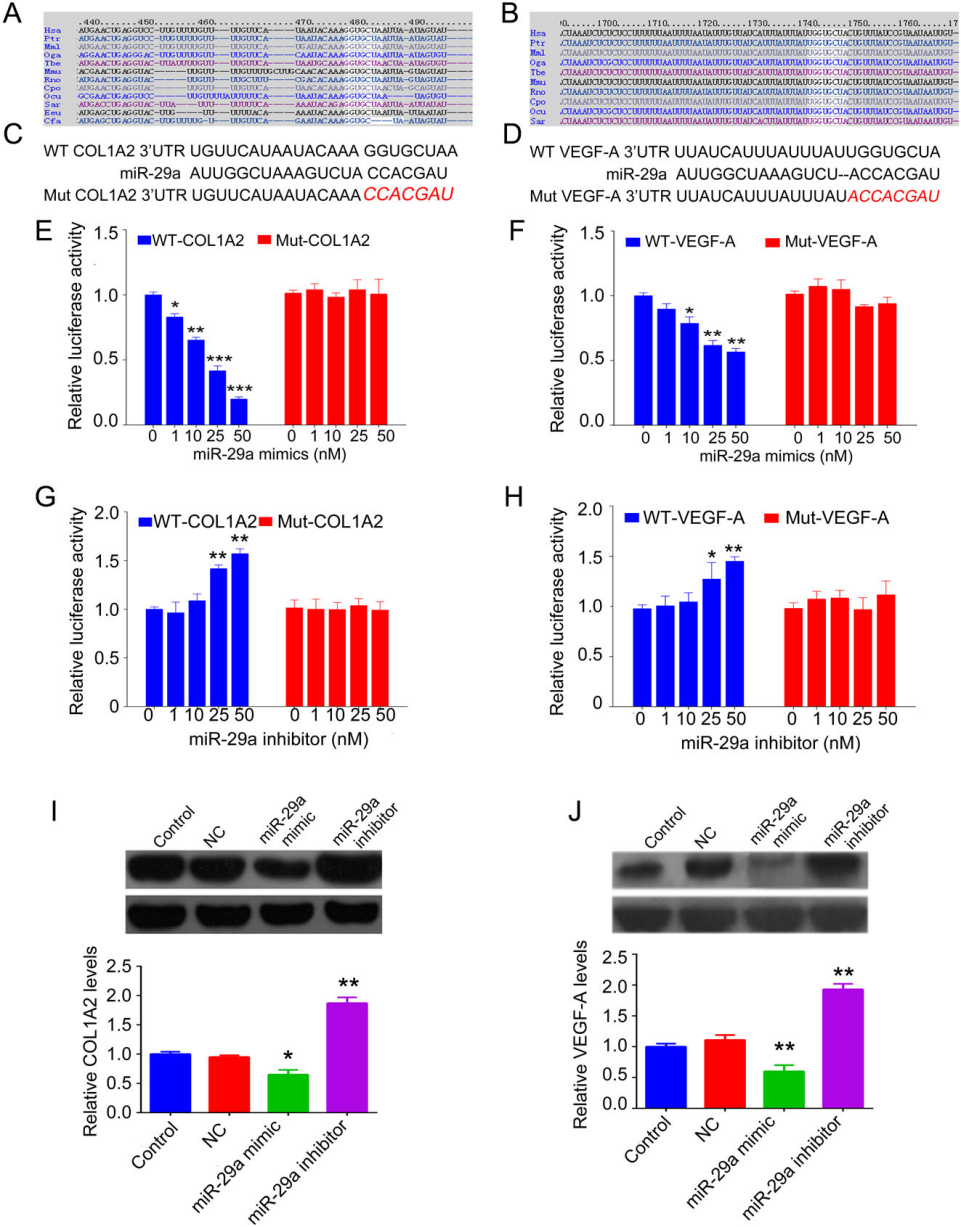
**COL1A2 and VEGF were negatively mediated by miR-29a in BJ cells.** (A) The predicted binding sites of COL1A2 to miR-29a in different species. (B) The predicted binding sites of VEGF-A to miR-29a in different species. (C) A wild-type (WT) and a mutant type (Mut, indicated by red italics) of COL1A2 3′ UTR as well as the putative seed sequences of miR-29a are shown. (D) A wild-type (WT) and a mutant type (Mut, indicated by red italics) of VEGF-A 3′ UTR as well as the putative seed sequences of miR-29a are shown. (E,G) A luciferase reporter assay was performed to determine whether COL1A2 is a target of miR-29a. A wild-type (WT) or mutant type (Mut) of COL1A2 3′ UTR was subcloned into the psiCHECK™2 luciferase miRNA expression reporter vector. PsiCHECK™-COL1A2-3′ UTR or psiCHECK™2-mut COL1A2-3′ UTR vector plus various concentrations of miR-29a mimics (E) or miR-29a inhibitor (G) were co-transfected into human skin fibroblast BJ cells. (F,H) A luciferase reporter assay was performed to determine whether VEGF-A is a target of miR-29a. A wild-type (WT) or mutant type (Mut) of VEGF-A 3′ UTR was subcloned into the psiCHECK™2 luciferase miRNA expression reporter vector. PsiCHECK™- VEGF-A-3′ UTR or psiCHECK™2-mut VEGF-A-3′ UTR vector plus various concentrations of miR-29a mimics (F) or miR-29a inhibitor (H) were co-transfected into human skin fibroblast BJ cells. (I-J) Western blot assay was performed to quantify the protein expression of COL1A2 (I) and VEGF-A (J) in BJ cells transfected with scramble miR (NC), miR-29a mimics, or miR-29a inhibitor, respectively. GAPDH was used as an internal reference. Control, BJ cells without any transfection; NC, cells transfected with blank vector. *P<0.05, **P<0.01 vs Control. The experiments were independently repeated for three times. Data are presented as mean±s.d.

As collagens are major components of denatured dermis and play a key role in tissue remodeling after thermal injury, we then focused on the identification of miR-29a-targeting genes associated with collagens synthesis. TargetScan was applied to obtain putative targets of miR-29a. As shown in Fig. 4A,B, COL1A2 and VEGF-A contained a conserved binding site of miR-29a in different species, suggesting that COL1A2 and VEGF-A were putative target genes. To further verify whether miR-29a can directly bind to their seed sequences in the 3′-UTR of COL1A2 and VEGF-A in BJ cells, we generated wild-type (WT) and mutant (MUT) constructs of COL1A2 3′ UTR and VEGF-A 3′ UTR (Fig. 4C,D). On performing a luciferase reporter assay, we showed that luciferase activity was significantly reduced in cells co-transfected with the wild-type COL1A2 or VEGF-A 3′ UTR and increasing concentrations of miR-29a mimics. Conversely, we found that miR-29a inhibitor (25 and 50 nM) significantly induced luciferase activity in cells transfected with wild-type COL1A2 or VEGF-A 3′ UTR; however, the luciferase activity showed no difference between cells co-transfected with mutant COL1A2 or VEGF-A 3′ UTR and miR-29a mimics (or miR-29a inhibitor), when compared to the control group (Fig. 4E-H). After upregulation of miR-29a, the protein levels of COL1A2 and VEGF-A were notably reduced, while miR-29a knockdown led to significant upregulation of COL1A2 and VEGF-A protein levels (Fig. 4I,J). These findings indicate that COL1A2 and VEGF-A are two direct targets of miR-29a, and the protein expression of COL1A2 and VEGF-A was negatively mediated by miR-29a in BJ cells.

### MiR-29a plays an inhibitory role in the proliferation and migration of BJ cells after thermal injury

**Fig. 5.**
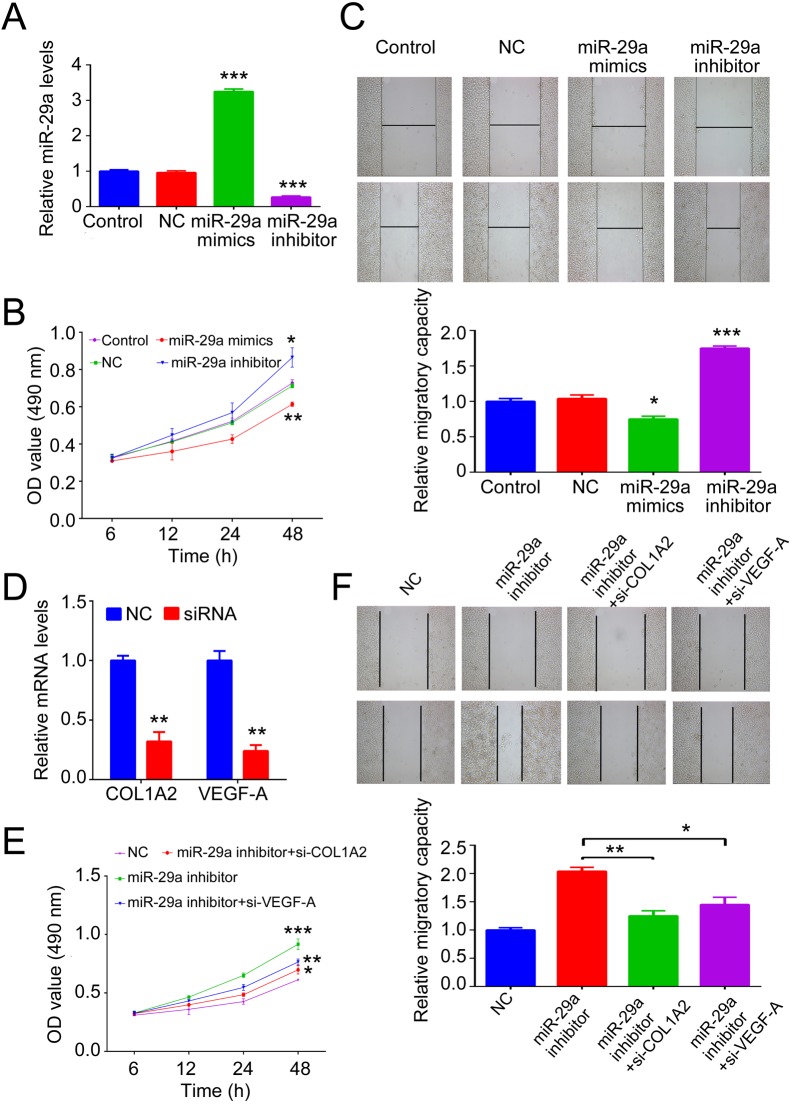
**Inhibitory role of miR-29a on proliferation and migration of BJ cells after thermal injury.** (A) Real-time RT-PCR was performed to examine the relative miR-29a expression in human skin fibroblast BJ cells transfected with scramble miR (NC), miR-29a mimics, or miR-29a inhibitor. Control: BJ cells without any transfection. ***P<0.001 vs Control. (B) MTT assay was performed to examine the proliferation capacity of BJ cells transfected with scramble miR (NC), miR-29a mimics, or miR-29a inhibitor, respectively, after thermal injury. **P<0.01 vs Control. (C) A wound healing assay was performed to examine the migratory capacity of BJ cells transfected with scramble miR (NC), miR-29a mimics, or miR-29a inhibitor, respectively, after thermal injury. *P<0.05, **P<0.01 vs Control. (D) Real-time RT-PCR was performed to examine the relative mRNA levels of *COL1A2* and *VEGF-A* in human skin fibroblast BJ cells transfected with negative control siRNA sequence (NC), siRNA *COL1A2* sequence (si-COL1A2), or siRNA *VEGF-A* sequence (si-VEGF-A). **P<0.01 vs NC. (E) An MTT assay was performed to examine the proliferation capacity of BJ cells transfected with NC or miR-29a inhibitor, or co-transfected with miR-29a inhibitor and si-COL1A2, or co-transfected with miR-29a inhibitor and si-VEGF-A, respectively, after thermal injury. (F) A wound healing assay was performed to examine the migratory capacity of BJ cells transfected with NC or miR-29a inhibitor, or co-transfected with miR-29a inhibitor and si-COL1A2, or co-transfected with miR-29a inhibitor and si-VEGF-A, respectively, after thermal injury. *P<0.05, **P<0.01, **P<0.001 vs NC. The experiments were independently repeated for three times. Data are presented as mean±s.d.

We further investigated whether miR-29a played a role in fibroblast cells after thermal injury. After transfection of BJ cells with miR-29a mimics or inhibitor, we firstly determined the transfection effect. As demonstrated in Fig. 5A, transfection with miR-29a mimics significantly led to increased miR-29a level in BJ cells compared to the control group without any transfection. By contrast, transfection with miR-29a inhibitor resulted in reduced miR-29a expression in BJ cells. Afterwards, thermal injury was conducted in BJ cells in each group, followed by performance of an MTT assay to determine cell proliferation. As shown in Fig. 5B, upregulation of miR-29a notably suppressed BJ cell proliferation, while knockdown of miR-29a promoted cell proliferation, when compared with that in the control group. We further investigated the effects of miR-29a upregulation or knockdown on the cell migration in BJ cells after thermal injury. Our findings showed that miR-29a overexpression notably inhibited cell migration, while downregulation of miR-29a significantly promoted cell migration, when compared to the control group (Fig. 5C). Furthermore, we also determined whether the effect of miR-29a on BJ cell proliferation and migration was via COL1A2 and VEGF-A. We knocked down the mRNA levels of *COL1A2* and *VEGF-A* (Fig. 5D). After 48 h of co-transfection with siRNA *COL1A2* or siRNA *VEGF-A* plasmid and miR-29a inhibitor in BJ cells, thermal injury was conducted in BJ cells in each group. Then, we performed an MTT assay to determine cell proliferation. As shown in Fig. 5E, COL1A2 and VEGF-A knockdown reversed miR-29a inhibitor-promoted cell proliferation compared to that in the control group. In addition, our findings also showed that the effect of miR-29a inhibitor on cell migration was rescued by *COL1A2* and *VEGF-A* siRNA transfection compared to the control group (Fig. 5F). Taken these findings together, we suggest that miR-29a plays an inhibitory role in the proliferation and migration of BJ cells via COL1A2 and VEGF-A after thermal injury.

### MiR-29a regulates Notch2, Jagged2 and MMP7 expression

**Fig. 6.**
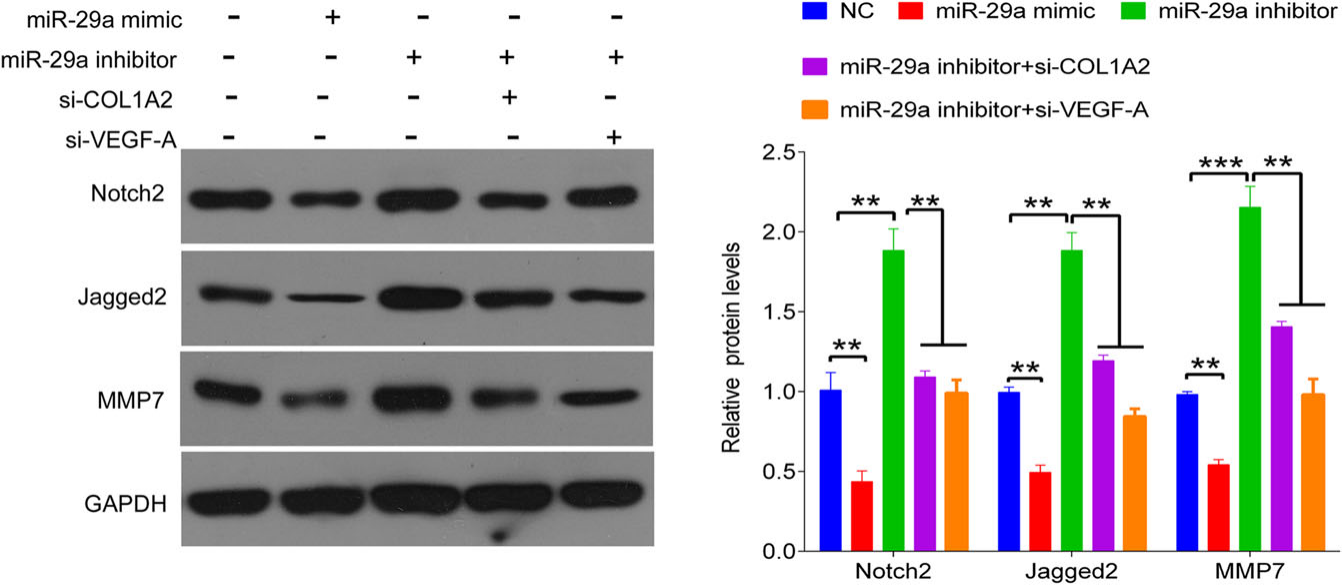
**MiR-29a inactivates Notch2 signaling.** Western blot assay was performed to quantify the protein expression of Notch2, Jagged2 and MMP7 in BJ cells transfected with scramble miR and negative control siRNA sequence (NC), miR-29a mimics, or miR-29a inhibitor, or co-transfected with miR-29a inhibitor and si-COL1A2, or co-transfected with miR-29a inhibitor and si-VEGF-A, respectively. GAPDH was used as an internal reference. The experiments were independently repeated for three times. Data are presented as mean±s.d., **P<0.01, ***P<0.001 vs NC.

To explore the potential downstream molecular pathway underlying miR-29a targeting to COL1A2 and VEGF-A, we tested the expression of proteins encoded by proliferation- and invasion-related genes, including Notch2, Jagged2 and MMP7, by western blot in BJ cells after transfection with miR-29a mimics or inhibitor. As shown in Fig 6, we observed a significant decrease in expression of Notch2, Jagged2 and MMP7 proteins in cells transfected with miR-29a mimics. Conversely, knockdown of miR-29a significantly increased the expression of Notch2, Jagged2 and MMP7 proteins in BJ cells compared to that of the control, while COL1A2 and VEGF-A knockdown reversed miR-29a inhibitor-mediated upregulation of Notch2, Jagged2 and MMP7 protein compared to that of the miR-29a inhibitor group.

## DISCUSSION

The denatured dermis, rich in collagen and appendages, has been demonstrated to play a critical role in the healing of burn injury via providing support and nourishment to the skin (Zhao et al., 2013). However, the role of miRs in denatured dermis during burn wound healing remained largely unclear. In the present study, we found that the expression of miR-29a was notably upregulated in denatured dermis tissues and skin fibroblast cells shortly after thermal injury, and thereafter gradually downregulated, accompanied by reverse expression profiles of its target genes, COL1A2 and VEGF-A, which have been implicated in wound healing. Moreover, we found that inhibition of miR-29a expression promoted the proliferation and migration of skin fibroblast cells after thermal injury. These findings suggest that miR-29a may play an important role in tissue remodeling after thermal injury, probably via mediating the proliferation and migration of skin fibroblast cells, as well as regulating the productions of type I collagen and VEGF-A.

Differential expression profiling of miRs has been demonstrated between mid-and late-gestational fetal skins, which are involved in the phenotypic transition from scarless to scarring repair during skin development, suggesting that miRs may play roles in scarless wound healing (Cheng et al., 2010). Yi et al. (2006) also suggest that discrete sets of differentially expressed miRs act as key regulators in skin morphogenesis in skin. Our previous study has compared the expression profile of miRs between the denatured dermis after burn injury and paired normal skin in human, and found that miR-29a was significantly downregulated in denatured dermis at day 4 after burn (Liang et al., 2012). To further investigate the role of miR-29a in burn wound healing, we constructed a rat model of thermal injury, and examined the expression profile of miR-29a in denatured dermis at different time points after thermal injury. A rapid upregulation of miR-29a was found shortly after thermal injury; however, with time, miR-29a was gradually downregulated. In the later phase of wound healing, its expression level was only ∼20% of the control group level. We speculated that the downregulated miR-29a might help wound healing.

To verify this speculation, we constructed a fibroblast cell model of thermal injury, as fibroblast cells are the major type of cells in denatured dermis, and play crucial roles in tissue repair and wound healing through mediation of collagen production and angiogenesis (Han et al., 2012; Newman et al., 2011; Zhao et al., 2015). We found a similar expression trend of miR-29a in fibroblast cells after thermal injury. More interestingly, inhibition of miR-29a notably enhanced fibroblast cell proliferation and migration shortly after thermal injury, suggesting that miR-29a is indeed involved in the healing of burn injury.

As the functions of miRs are mainly through mediating the expression of their target genes, we further focused on the targets of miR-29a, which may contribute to the recovery of denatured dermis function after thermal injury. Our findings showed that COL1A2 was identified as a direct target of miR-29a, and miR-29a negatively mediated the expression level of COL1A2 in fibroblast cells. COL1A2 encodes the pro-alpha2 chain of type I collagen whose triple helix comprises two alpha 1 chains and one alpha 2 chain (Reuter et al., 2013). Type I is a fibril-forming collagen found in most connective tissues and is abundant in bone, cornea, dermis and tendon (Trojanowska et al., 1998). Improvement of type I collagen production is critical for the healing of burn injury (Newman et al., 2011). Interestingly, the expression profiling of COL1A2 was opposite to that of miR-29a in denatured dermis and fibroblast cells at different times points after thermal injury.

Angiogenesis is a crucial process for the formation of new blood vessels. Through providing oxygen, nutrients and various growth factors to sites of tissue repair, angiogenesis is critical to the recovery of heat denatured dermis (Lancerotto and Orgill, 2014; Liang et al., 2013). VEGF-A, which has been identified as a direct target of miR-29a, acts as a pro-angiogenic factor secreted by fibroblast cells (Chen et al., 2014). Moreover, promotion of VEGF-A expression can help improve wound healing (Mirza and Koh, 2015). In our study, we showed that the expression profiling of VEGF-A, similar to COL2A1, was also opposite to that of miR-29a in denatured dermis and fibroblast cells at different times points after thermal injury. Taken together, these findings suggest that the role of miR-29a in the healing of burn injury is at least partly through its mediation of COL2A1 and VEGF-A expression in fibroblast cells.

Our results also shows that miR-29a regulates Notch/Jagged signaling via its targets, COL2A1 and VEGF-A. Notch signaling is also involved in regulating cell fate and maintaining skin homeostasis (Bielefeld et al., 2013). Increasing evidence suggests that aberrant Notch signaling may contribute directly to skin pathogenesis and altered expression of Notch receptors (Syed and Bayat, 2012). Transgenic mice expressing a Notch antisense sequence exhibit delayed healing; while mice treated with the Notch ligand, Jagged, show accelerated wound closure, suggesting that these effects are mediated by the Notch pathway (Chigurupati et al., 2007). By contrast, Notch heterozygous mice exhibit increased collagen deposition and vascularity in healing wounds, and Notch1 can modulate VEGF1 expression and matrix-adhering involved in matrix metalloprotease, such as MMP7 (Caiado et al., 2008; Outtz et al., 2010). The experiments *in vitro* also show that the Notch/Jagged pathway involves pro-migratory effects on fibroblast and vascular endothelial cells (Chigurupati et al., 2007). There is evidence that miR-29a participation in wound repair may be via the Notch pathway, which in turn may be involved in several aspects of healing, such as angiogenesis and matrix production.

Besides miR-29a, other miRs have also been suggested to be associated with wound healing in skin. For instance, miR-21 regulates skin wound healing by targeting multiple aspects of the healing process including wound contraction and collagen deposition (Wang et al., 2012). In addition, miR-27b was showed to prolong burn wound repair, by inhibiting the migration of mesenchymal stem cells to burned margins through silencing the expression of stromal cell-derived factor-1α (Lu et al., 2013).

In conclusion, the present study suggests for the first time an important role of miR-29a in the healing of thermal injury. Inhibition of miR-29a can promote not only the proliferation and migration of skin fibroblast cells after thermal injury, but also the production of COL1A2 and VEGF-A, which can further enhance the collagen synthesis and angiogenesis in skin.

## MATERIALS AND METHODS

### Rat model of thermal injury

All rats used in this study were purchased from the Laboratory Animal Center of Central South University (Changsha, China). Animals were housed in separate cages in a temperature-controlled room with 12 h light and 12 h darkness, and had free access to water. All experiments in our study were in compliance with the guide for the care and use of laboratory animals of Central South University. A deep partial-thickness burn model in SD rats was established as previously described (Liang et al., 2012). Briefly, 6 rats for each group were anesthetized with 10% chloralhydrate (0.5 ml/100 g). The backs of the rats were shaved with an electrical clipper. An aluminum cylinder (3.76 cm in diameter, 3.78 cm in height) was placed into 90°C water for 15 min and pressed on the back of rats for 15 s to produce a deep partial-thickness burn wound, which was confirmed by pathological examination. The effective wound diameter was 2.5 cm. Denatured dermis was harvested following anesthetizing rats with 10% overdose chloralhydrate (0.5 ml/100 g) at days 1, 3, 5 and 7 after burn creation and then rats were killed by decapitation. The rats in the control group were given the same treatment but were exposed to the cylinder at room temperature. The isolated skin tissues were immediately frozen in liquid nitrogen and stored at −80°C for further analyses.

### Haematoxylin and eosin staining

Skin specimens were fixed in 4% paraformaldehyde solution in phosphate buffer overnight. These samples were bisected in the sagittal plane through the center and embedded in paraffin, and subsequent serial sections (16 mm in thickness) were cut on a cryostat and mounted onto coated glass slides. Haematoxylin and eosin (HE) staining was performed to evaluate the structural features and cellular morphology.

### Cell culture

Human skin fibroblast BJ cell line was purchased from China Center for Type Culture Collection (Wuhan, China). BJ cells were cultured in DMEM supplemented with 10% fetal bovine serum (FBS, Life Technologies, Carlsbad, CA, USA), 100 IU/ml penicillin, and 100 μg/ml streptomycin sulfate at 37°C in a humidified incubator containing 5% CO_2_.

### Skin fibroblast cell model of thermal injury

Human skin fibroblast BJ cells in each group were digested and suspended in 10 ml DMEM with 10% FBS. Then, the cell suspension was incubated in 52°C water for 30 s. In the control group, the suspension of BJ cells was incubated in 37°C water for 30 s. After that, cells were further cultured at 37°C in a humidified incubator containing 5% CO_2_.

### Real-time RT-PCR

For mRNA expression detection, total RNA was extracted from tissues or cells by using Trizol reagent (Life Technologies) following the manufacturer's instructions. The expression of mRNA was detected by real-time RT-PCR using the standard SYBR Green RT-PCR Kit (Takara, Otsu, Japan) following the manufacture's instructions. The specific primer pairs are as follows; COL1A2 sense: 5′-GTTGCTGCTTGCAGTAACCTT-3′, antisense: 5′-AGGGCCAAGTCCAACTCCTT-3′; VEGF-A sense: 5′-AGGGCAGAATCATCACGAAGT-3′, antisense: 5′-AGGGTCTCGATTGGATGGCA-3′; GAPDH as an internal control, sense: 5′-GGAGCGAGATCCCTCCAAAAT-3′, antisense: 5′-GGCTGTTGTCATACTTCTCATGG-3′. For miR expression detection, miR was isolated from cells by MiRNeasy Mini Kit (Qiagen, Valencia, CA, USA), according to the manufacture's instructions. MiRNA reverse transcription kit (Life Technologies) was used to convert RNA into cDNA, according to the manufacturer's instructions. The expression of miRNA was then determined using the TaqMan MicroRNA Assays Kit (Life Technologies) on a 7500 Fast Real Time PCR System (Life Technologies). U6 was used as an endogenous reference. The relative expression of mRNA or miRNA was quantified using GraphPad Prism 4.0 software (GraphPad Software, San Diego, CA, USA) and 2^−ΔΔCt^ method.

### Western blotting assay

Cells were lysed in cold RIPA buffer (Life Technologies). The BCA Protein Assay Kit (Life Technologies) was used to determine the protein concentration. Protein was then separated with 10% SDS-PAGE, and transferred to a PVDF membrane. The PVDF membrane was blocked in 5% nonfat dried milk in PBS for 4 h. After that, the PVDF membrane was incubated with the following primary antibodies for 3 h: rabbit polyclonal anti-COL1A2 (cat no. ab208638; 1:400), mouse monoclonal anti-VEGF-A (cat no. ab155944; 1:100), rabbit polyclonal anti-Notch2 (cat no. ab137665; 1:500), rabbit monoclonal anti-Jagged2 (cat no. ab109627; 1:5000), rabbit monoclonal anti-MMP7 (cat no. ab205525; 1:4000), mouse monoclonal anti-GAPDH antibody (cat no. ab181602; 1:200). All the antibodies were purchased from Abcam, Cambridge, UK. After washing with PBS three times for 5 min, the PVDF membrane was incubated with the rabbit anti-mouse secondary antibody (1:20,000; all antibodies were purchased from Abcam, Cambridge, UK). After washing with PBS three times for 5 min, an ECL Western Blotting Kit (Millipore, Darmstadt, Germany) was used to detect the immune complexes on PVDF membrane.

### Dual luciferase reporter assay

Wild-type (WT) and mutant (MUT) forms of the 3′ UTR of COL1A2 or VEGF-A were inserted downstream of the dual luciferase reporter vector. For the luciferase assay, 5×10^4^ BJ cells were plated and cultured in 96-well plates to reach approximately 80% confluence. The cells were co-transfected with a range of concentrations (0, 1, 10, 25, 50 nM) of miR-29a mimics or miR-29a inhibitors and 25 ng of the WT/MUT 3′ UTR of COL1A2 or VEGF-A dual luciferase reporter vector using Lipofectamine 2000 (Life Technologies, Carlsbad, CA). After 48 h of transfection, a Dual-Luciferase Reporter Assay System (Promega, Madison, WI, USA) was used to detect luciferase activity using a GloMax^®^-Multi+ Luminometer (Promega). Luciferase activity was normalized to Renilla luciferase activity.

### Cell proliferation assay

An MTT assay was used to measure cell proliferation. Cells in each group were cultured in 96-well plates, each well with 100 μl of fresh serum-free medium with 0.5 g/l MTT. After incubation at 37°C for 6, 12, 24, and 48 h, the medium was removed by aspiration and 50 μl of DMSO was added to each well. After incubation at 37°C for a further 10 min, the A492 of each sample was measured using a plate reader.

### Wound healing assay

A wound healing assay was performed to evaluate the cell migratory capacity of BJ cells transfected with scramble miR (NC), miR-29a mimics, or miR-29a inhibitor (Nlunbio Company, Changsha, China), respectively, after thermal injury. In brief, cells were cultured to full confluence. Wounds of approximately 1 mm width were created with a plastic scriber, and cells were washed and incubated in a serum-free medium. Cells were incubated in a medium including 10% fetal bovine serum for 24 h after wounding. After further cultures for 0 and 48 h, cells were fixed and observed under a microscope.

### Statistical analysis

Data are expressed as mean±s.d. of three independent experiments. The differences between groups were determined using one-way ANOVA. The correlation between miR-29a and COL1A2 or VEGF-A was analyzed using a Pearson correlation analysis. Statistical analysis was performed by using SPSS 18.0 statistical software (SPSS, Chicago, IL, USA). *P<0.05 was considered statistically significant.
